# Application of High-Efficiency Cell Expansion and High-Throughput Drug Sensitivity Screening for Leukemia Treatment

**DOI:** 10.1155/2022/4052591

**Published:** 2022-07-05

**Authors:** Lili Li, Wenliang Wang, Li Liang, Jian Ge, Ruixiang Xia

**Affiliations:** ^1^Department of Hematology, The First Affiliated Hospital of Anhui Medical University, Hefei, Anhui 230022, China; ^2^Precision Targeted Therapy Discovery Center, Institute of Technology Innovation, Hefei Institutes of Physical Science, Chinese Academy of Sciences, Hefei, Anhui 230088, China

## Abstract

This study is to assess the clinical value of in vitro primary cell high-efficiency expansion and high-throughput drug sensitivity screening (HEHDS) system in leukemia, and we evaluated a cohort of 121 patients with acute myeloid leukemia (AML) and 27 patients with acute lymphoblastic leukemia (ALL) using HEHDS. Bone marrow aspirates were collected from patients with leukemia. Purified leukemic cancer cells were obtained, cultured, and screened with a panel of 247 FDA-approved compounds by HEHDS technology. Ninety-six patients received HEHDS-guided therapy while 52 patients who were subjected to physician directed therapy served as controls. ALL patients who received treatment guided by HEHDS showed higher rate of complete remission (CR) than that of patients in the non-HEHDS group (90.91% vs. 56.25%). Similarly, AML patients received HEHDS-guided therapy were found to have greater CR rate, when compared with patients who received physician-directed therapy (45.88% vs. 25%). There was a significantly higher rate of CR in HEHDS-guided therapy group compared to the non-HEHDS group. The application of HEHDS could be beneficial for leukemia treatment.

## 1. Introduction

Leukemia is a blood cancer that leads to high death rate worldwide [1–3]. To date, chemotherapy is the main treatment of leukemia with promising efficacy [4–6]. However, due to the heterogeneity of leukemia cells, no regimen has been shown to be superior in most cases [7, 8]. There are problems including increased complications, high recurrence rate, and reduced immunity as well as unsolved thrombocytopenia [9]. The effectiveness of commonly used antitumor chemotherapy drugs for patients is less than 70%, and 20%-40% of patients may even receive the inappropriate regimens. Therefore, chemotherapy drugs on an individual level can be a feasible methodology to improve the efficiency of chemotherapy and reduce the occurrence of multidrug resistance [10].

As a part of the precision medicine program, drug sensitivity screening has been more and more widely applied in recent years [11, 12]. Although sensitive drugs of all the cancers could be screened theoretically, factors like finite cell number, the lack of tumor biopsy, and the high degree of heterogeneity in the tumor often result in unsatisfied clinical outcomes. As such, many studies mainly focused on liquid cancer, such as leukemia [13–16]. However, isolated leukemia cells are only enough for a small amount of drug screening. Moreover, the detection flux of traditional drug sensitivity assay is low, and the number of drugs that can be detected is limited [17]. Therefore, we want to culture leukemia cells on a large scale in vitro and conduct large-scale drug screening, so that we can help patients to find more effective drugs and suggest suitable therapeutic approach for patients at high risk.

In present study, we explore whether high-efficiency expansion and high-throughput drug sensitivity screening (HEHDS) can overcome the limitations of previous technologies that only screen a small number of drugs and improve the clinical treatment effect of leukemia. *In vitro* drug sensitivity screening from cells derived from leukemia patients was performed, and the efficacy of therapy under the guidance of HEHDS was evaluated. Our findings herein imply the potential of HEHDS as a personalized treatment strategy for patients with hematological diseases.

## 2. Materials and Methods

### 2.1. Patients

A total of 121 patients with AML (except M3 type) and 27 patients with ALL admitted to our department from May 2008 until May 2020 were included in this study. Patients were randomly divided into HEHDS-guided therapy group and non-HEHDS group. HEHDS-guided therapy group received high-throughput drug sensitivity analysis in order to select efficacious drugs. Personalized treatment strategies were generated according to screening results and continued until disease progression or unacceptable toxicity occurred. For the non-HEHDS group, HEHDS was performed but the patients were still treated with standard chemotherapy that instructed by clinicians. The clinical samples used for drug sensitivity test were collected before treatment. Gene mutations, chromosomes, and other samples of the included patients were collected before treatment. Clinical samples for evaluating the efficacy were collected from the 21^st^ day to the 28^th^ day after the end of chemotherapy.

All patients provided written informed consent. The study was approved by the ethics committee of the First Affiliated Hospital of Anhui Medical University and complied with the ethical guidelines of the Helsinki Declaration, revised in 1975.

### 2.2. Diagnosis and Treatment Standards

All patients were diagnosed based on MICM (cell morphology, immunology, genetics, and molecular biology) Classification criteria according to the 2016 revision to the World Health Organization classification of myeloid neoplasms and acute leukemia. All patients were treated based on guidelines for the diagnosis and treatment of adult acute myeloid leukemia (2018 edition) [18–20].

### 2.3. Inclusion Criteria

(1) Newly diagnosed patients: according to the WHO (2016) classification criteria for hematopoietic and lymphoid tissue tumors, the proportion of blast cells in peripheral blood or bone marrow of patients is greater than or equal to 20%. Or the bone marrow blast ratio is less than 20%, coexistence of clonal recurrent cytogenetic abnormalities t(8;21)(q22;q22), inv(16)(p13q22) or t(16;16)(p13;q22), and t(15;17)(q22;q12). (2) Relapsed patients: after the patient achieves complete remission, leukemia cells or bone marrow blasts > 5% or extramedullary hemorrhage leukemia cell infiltration occurs again in the peripheral blood. (3) Refractory patients: these are newly treated cases that are ineffective after 2 courses of standard regimens. The patients achieved CR and relapsed within 12 months after consolidation and intensive therapy and relapsed after 12 months but ineffective after conventional chemotherapy. These are patients with 2 or more recurrences and patients with persistent extramedullary leukemia. (4) Patients aged ≥l14 years. (5) Patients received a full course of chemotherapy. During the same study period, we observed a total of 170 patients, of whom 148 were included in the study and 22 were not eligible.

### 2.4. Isolation and Culture of Leukemia Cells

Peripheral blood mononuclear cell (PBMC) samples of patients were collected. Mononuclear cells from blood samples were separated using Percoll-P4937 (Sigma-Aldrich, St. Louis, MO, USA) according to manufacturer's instructions. Red blood cells are removed by Red cell lysis buffer (Sigma-Aldrich, St. Louis, MO, USA). The leukemia cell pellets were washed with phosphate-buffered saline (PBS), resuspended, and cultured with CM medium (Precedo, Hefei, China).

### 2.5. Leukemia Cell Proliferation Measurement

The proliferation rate of each generation was counted and calculated according to the formula: population doubling (PD) = 3.32∗log_10_ (C1/C0), where C1 is the number of harvested cells and C0 is the number of initial cells. The rest leukemia cells were used for drug sensitivity screening.

### 2.6. High-Throughput Drug Sensitivity Screening

For HEHDS-guided therapy, leukemia cells were cultured with complete medium (Precedo, Hefei, China) at 37°C in 5% CO_2_ incubator. The high-throughput drug sensitivity screening was performed as previously reported [21]. To assess drug susceptibility, cell plating was carried out by counting cells in the logarithmic growth phase and then adding them in a white 384-well cell culture plate with a density of 1 × 10^5^ cells/ml. Cell suspension (50 *μ*L) was added to each well. All drugs were dissolved and diluted using dimethylsulfoxide (DMSO). The concentrations were those used in clinical practice and met the international drug standard. The control group was treated with DMSO. Drug (0.1 *μ*L) was added in plate using a JANUS automated workstation (Perkin Elmer Inc., Wellesley, MA, USA). The cells were incubated for 72 h, and then, 10 *μ*L CellTiter-Glo cell proliferation fluorescence detection reagent was added to each well. Fluorescence was measured using an EnVision plate reader (Perkin Elmer Inc., Wellesley, MA, USA). The results were analyzed using the following equation: [inhibition rate = 100% − (RLU_Drug_ − RLU_Background_)/(RLU_DMSO_ − RLU_Background_) × 100%]. Drugs with inhibition rate greater than 70% were defined as sensitive candidates and selected for treatments according to the medication history of patients. The drug library mainly includes 30 traditional Chinese medicine monomers, 65 targeted drugs, 107 chemotherapy drugs, and 45 combination regimens.

### 2.7. Data Analysis

Statistical analysis was performed using SPSS 10.0 (IBM Corp., Armonk, NY, USA). Fisher's exact test was used in order to detect the factors that influenced the CR rate. *P* values reported are two-sided, and *P* value < 0.05 was considered to be statistically significant in both groups.

## 3. Results

### 3.1. Patient Characteristics

A total of 27 ALL patients and 121 AML patients were enrolled in our study (Table 1). The median age of ALL patients including 13 males and 14 females was 37 years (16-77 years). There were 11 newly diagnosed and 16 relapsed patients. Eighteen of them belonged to normal karyotype without any molecular abnormality except 9 patients that had BCR-ABL mutation. Eleven patients were selected for HEHDS-guided therapy whereas the rest were treated with physician-directed therapy. By contrast, AML patients consisted of 57 females and 64 males. The median age of these 43 newly diagnosed and 78 relapsed patients was 48 years (13-77 years). There were 23 patients who had FLT3-ITD mutation. Overall, 85/121 patients received HEHDS-guided therapy, and 36/121 patients were treated with physician-directed therapy in the AML group.

### 3.2. *In Vitro* Culture of Leukemia Cells and Drug Sensitivity Screening

**Figure 1:**
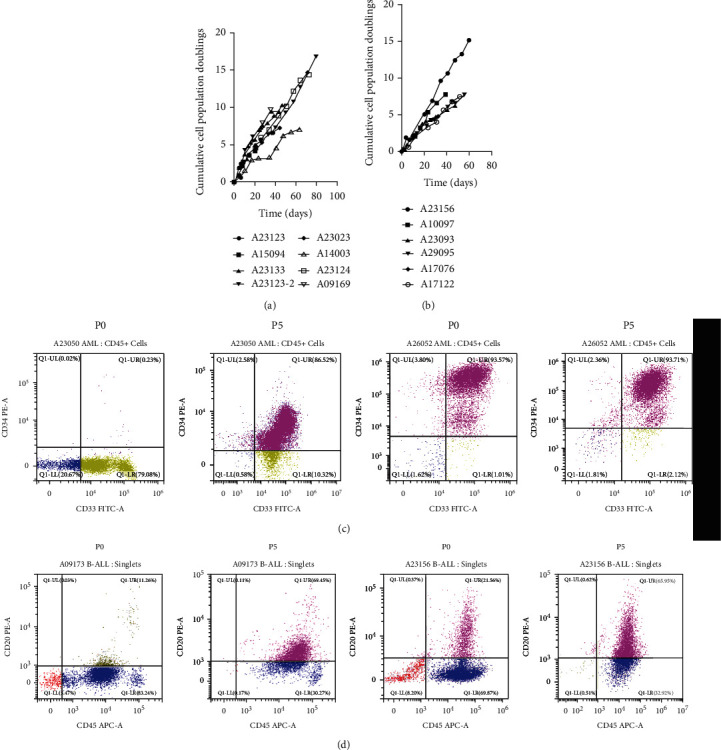
*In vitro* culture of leukemia cells. (a) AML cells obtained from eight cohorts were cultured continuously, and the growth rate of each generation was recorded. (b) ALL cells obtained from six cohorts were cultured continuously, and the growth rate of each generation was recorded. (c) The AML cell surface markers (CD33 and CD34) of P0 and P5 generations were analyzed by FACS analysis. (d) The ALL cell surface markers (CD45 and CD20) of P0 and P5 generations were analyzed by FACS analysis.

*In vitro* drug sensitivity testing is one of the effective methods for precise tumor treatment. However, due to the limited number of tumor cells obtained, most samples can only detect the sensitivity of a small number of drugs. This makes it difficult for most patients to find drugs that are truly suitable. To overcome the limitation of cell number obtained from samples for drug sensitivity screening, we have successfully developed a set of culture system suitable for tumor cell growth *in vitro*. Using this culture system, leukemia cells could be cultured *in vitro* for long term (Figures 1(a) and 1(b)). As analyzed by FACS, the cultured cells still showed their identity as AML or ALL derived after 5 passages (Figures 1(c) and 1(d)). By combining leukemia *in vitro* amplification and drug susceptibility technology, we tested 247 different drugs and multidrugs for all patients in order to identify personalized drugs for better clinical outcomes.

### 3.3. Response and Treatment Outcome

Subsequently, 27 patients were categorized into HEHDS-guided therapy group (*n* = 11) and non-HEHDS group (*n* = 16). As shown in Table 2, the total CR rate of 11 ALL patients received treatment guided by HEHDS was 90.91% and that of 16 patients in the non-HEHDS group was 56.25%. For patients with new diagnosed ALL, the CR rate of the HEHDS-guided group was 100%, which was significantly higher than that of the non-HEHDS group (50%). For patients with relapsed ALL, the CR rate of the HEHDS-guided group was 75% and the rate of the non-HEHDS group was 58.3% in relapsed patients. Overall, the CR rate of 85 AML patients in the HEHDS-guided therapy group was 45.88%, and that of 36 patients in the non-HEHDS group was 25%. Compared with patients in the non-HEHDS group, the CR rate was significantly higher for the HEHDS-guided group with newly diagnosed AML (54.55% vs. 20%). Similarly, the CR rate of the HEHDS-guided group (40.1%) was also higher than that of the non-HEHDS group (26.92%) in relapsed AML (Table 3).

### 3.4. Analysis of Factors Influencing HDS-Guided CR in AML

Gene mutations are common in AML, and genes such as TP53, KIT, RAS, and FLT3-ITD contribute to the development of AML. These mutations are often associated with poor prognosis. In this study, we found that among 5 patients with TP53 mutations, the CR rate of 3 patients receiving HEHDS-guided therapy was 66.67%, and that of 2 patients in the non-HEHDS group was 50%. In 12 patients with KIT mutations, the CR rate of 10 patients received HEHDS-guided therapy was 40% and it was 50% in 2 patients in the non-HEHDS group. In 20 patients with NRAS mutations, the CR rate of 14 patients received HEHDS-guided therapy was 28.57% and it was only 16.67% in 6 patients in the non-HEHDS group, suggesting that HEHDS-guided therapy could benefit patients with NRAS mutations. In support with previous studies, the prognosis of FLT3-ITD AML patients was poor. Surprisingly, we found that in this group, 17 patients received HEHDS-guided therapy had greater CR rate, when compared with those (*n* = 6) who received physician-directed therapy (47.06% vs. 16.67%) (Table 4). The data indicate that HEHDS-guided therapy may be able to improve CR in leukemia patients significantly, especially in FLT3-ITD AML patients. In addition, we also assessed the impact of WBC count on HEHDS-guided therapy. When WBC count was fewer than 50 × 10^9^/L prior to treatment, the CR rate of 64 patients received HEHDS-guided therapy was 45.3%, and it was only 31% in 29 patients in the non-HEHDS group. Compared with physician-directed therapy (*n* = 7), HEHDS-guided therapy dramatically improved the CR rate in 21 patients that have ≥50 × 10^9^/L WBC, with the figure increased from 0 to 47.6% (Table 4). Together, these results demonstrated that WBC could be a critical factor that influences the effectiveness of HEHDS-guided therapy in AML patients. We also assessed the impact of median marrow blast on HEHDS-guided therapy. Compared with the non-HEHDS group, whether median marrow blast is greater than (*n* = 22) or less than (*n* = 14) 10%, the CR of HEHDS-guided therapy has a certain improvement (*n* = 45 or 40).

### 3.5. HEHDS-Guided Therapy Enhances the Clinical Outcome of Combination Chemotherapy in AML

A combination of chemotherapy may greatly improve the therapeutic effect, and therefore, IA (idarubicin and cytarabine) and DA (daunorubicin and cytarabine) programs are widely used nowadays. However, none of them is universal to all patients. In current study, we investigated the effect of HEHDS on improving the response rate of IA and DA. It was found that in patients (*n* = 42) treated with IA, the CR rate of patients (*n* = 31) received HEHDS-guided therapy was 48% and that of the non-HEHDS group (*n* = 11) was 27%. Nevertheless, when treated with DA (*n* = 16), 38.46% of patients (*n* = 13) who received HEHDS-guided therapy achieved 38.46% CR. Although the CR rate was 33.3% in the non-HEHDS group, the data involved was too small (*n* = 3) to compare with that of the HEHD group (Table 5). The results indicated that HEHDS-guided therapy could improve the therapeutic effect of combination chemotherapy.

## 4. Discussion

Over the past decades, leukemia is a heterogeneous disease characterized by overt poor prognosis. Despite treated with high-dose chemotherapy and hematopoietic stem cell transplantation, most patients face a failure to achieve complete remission [22, 23]. Precision medicine could provide patient-specific treatment guidance to eliminate the need for cycles of chemotherapy which believed to overcome this issue [24]. *Ex vivo* drug sensitivity screening includes two steps: tumor cell isolation and drug sensitivity detection [25]. The characteristic of our approach is to expand tumor cells obtained from patients that are enough for drug sensitivity testing. At present, due to the limited number of leukemia cells isolated directly from patients, only few drugs could be tested immediately. To date though, there are various methodologies for culture of leukemia cells *in vitro*, and the cell proliferation rate could hardly meet the needs of large-scale test. The medium we utilized in current study not only supported the rapid expansion of primary leukemia cells but also ensured their biological characteristics similar as *in vivo*. Specifically, a 7-fold amplification rate was seen within one week.

To test the validity of HEHDS, patients were treated with either HEHDS-guided therapy or physician-directed therapy. Compared with the control group, the CR rate in the HEHDS-guided group was significantly higher in both ALL and AML cohorts. It shows that HEHDS-guided therapy is very effective in the guidance of clinical medication of leukemia. Most importantly, the result shows that HEHDS-guided therapy can significantly improve the CR rate of relapsed and refractory patients, of whom the median survival time is less than one year.

The presence of unfavorable gene mutations, such as FLT3-ITD, often relates to a poor response to clinical treatment [26–29]. In this study, we compared the effects of HEHDS-guided therapy and non-HEHDS therapy on CR rates in patients with some common gene mutations. The results showed that the CR rate of HEHDS-guided patients was significantly improved in patients with FLT3-ITD and NRAS mutations.

The IA and DA regimens are commonly used regimen in clinical practice that enhances the overall survival of patients [30, 31]. However, many patients fail to respond to these treatments. Predicting the response of patients to regimens in advance is critical to improve patient outcomes [32]. Hence, we examined the effect of HEHDS-guided therapy on the treatment efficiency of these regimens. Not surprisingly, patients treated by HEHDS-guided therapy achieved CR more easily following combination chemotherapy. Overall, HEHDS-guided therapy might benefit patients receiving induction chemotherapy.

### 4.1. Study Limitation

The number of samples in this study is limited, and further studies are needed to evaluate the application of HEHDS in leukemia treatment.

## 5. Conclusion

In summary, our study establishes a valuable approach that could effectively improve the CR rate in leukemia, providing a possible treatment strategy for patients at highest risk.

## Figures and Tables

**Table 1:** Patient characteristics.

Disease subtype	Characteristics		*N* (range)	HEHDS	Non-HEHDS guided	*P* values
AML	Patients		121	79	42	
	Male/female		57/64	35/44	22/20	0.397
	Median age		48	46	50	0.238
	Median marrow blast %		11.23%	11.00%	11.24%	0.325
	Genetic anomaly at initial diagnosis	Normal karyotype without any molecular abnormality	98	62	36	0.334
		FLT3-ITD	23	17	6	
	Disease status	New diagnosed	43	26	17	0.408
		Relapsed	78	53	25	

ALL	Patients		27	17	10	
	Male/female		13/14	7/10	6/4	0.345
	Median age		37	35	39	0.195
	Median marrow blast %		10.25%	9.57%	11.15%	0.251
	Genetic anomaly at initial diagnosis	Normal karyotype without any molecular abnormality	18	11	7	0.778
		BCR-ABL	9	6	3	
	Disease status	New diagnosed	11	7	4	0.952
		Relapsed	16	10	6	

**Table 2:** Complete remission analysis of ALL.

Characteristics	Total CR rate (%)	CR rate/non-HEHDS guided (%)	CR rate/HEHDS guided (%)	*P* values^1^
	19/27 (70.37)	9/16 (56.25)	10/11 (90.91)	0.062

New diagnosed	9/11 (81.82)	2/4 (50)	7/7 (100)	0.109
Relapsed	10/16 (62.5)	7/12 (58.3)	3/4 (75)	0.511
*P* value^2^	0.261	0.608	0.364	

^1^Non-HEHDS-guided CR rate vs. HEHDS-guided CR rate. ^2^The CR rate of new diagnosed vs. the CR rate of relapsed.

**Table 3:** Complete remission analysis of AML.

Characteristics	Total CR rate (%)	CR rate/non-HEHDS guided (%)	CR rate/HEHDS guided (%)	*P* values^1^
	48/121 (39.67)	9/36 (25)	39/85 (45.88)	0.025

New diagnosed	20/43 (46.51)	2/10 (20)	18/33 (54.55)	0.058
Relapsed	28/78 (35.9)	7/26 (26.92)	21/52 (40.1)	0.181
*P* value^2^	0.171	0.514	0.146	

^1^Non-HEHDS-guided CR rate vs. HEHDS-guided CR rate. ^2^The CR rate of new diagnosed vs. the CR rate of relapsed.

**Table 4:** Analysis of factors influencing HEHDS-guided CR in AML.

Factors		Total CR%	CR rate/non-HEHDS guided (%)	CR rate/HEHDS guided (%)	*P* values
Mutation	TP53	3/5 (60)	1/2 (50)	2/3 (66.67)	0.667
	KIT	5/12 (41.67)	1/2 (50)	4/10 (40)	0.682
	NRAS	5/20 (25)	1/6 (16.67)	4/14 (28.57)	0.517
	FLT3-ITD	9/23 (39.13)	1/6 (16.67)	8/17 (47.06)	0.208

Median WBC count	<50 × 10^9^/L	38/93 (40.9)	9/29 (31)	29/64 (45.3)	0.142
	≥50 × 10^9^/L	10/28 (35.7)	0/7 (0)	10/21 (47.6)	0.027

Median marrow blast %	<10	28/54 (51.9)	5/14 (35.7)	23/40 (57.5)	0.137
	≥10	20/67 (30)	4/22 (18.2)	16/45 (35.6)	0.119

**Table 5:** HEHDS-guided therapy enhances the clinical effect of drug combination in AML.

Treatment programs	Total CR (%)	CR rate/non-HEHDS guided (%)	CR rate/HEHDS guided (%)	*P* values
IA	18/42 (43)	3/11 (27)	15/31 (48)	0.196
DA	6/16 (37.5)	1/3 (33.33)	5/13 (38.46)	0.696

## Data Availability

The datasets used and/or analyzed during the current study are available from the corresponding author on reasonable request.
